# Discussion on the Timing of Balloon Occlusion of the Abdominal Aorta during a Caesarean Section in Patients with Pernicious Placenta Previa Complicated with Placenta Accreta

**DOI:** 10.1155/2017/8604849

**Published:** 2017-11-02

**Authors:** Baoju Zhu, Kaili Yang, Lina Cai

**Affiliations:** ^1^Department of Gynaecology and Obstetrics, The Second Affiliated Hospital of Zhengzhou University, Zhengzhou, Henan 450014, China; ^2^Department of Gynaecology and Obstetrics, People's Hospital of Zhengzhou, Zhengzhou, Henan 450014, China

## Abstract

**Objective:**

This paper is aimed at investigating the role and value of the timing of balloon occlusion of the abdominal aorta during caesarean section in patients with pernicious placenta previa complicated with placenta accreta.

**Methods:**

79 cases admitted to the Second Affiliated Hospital of Zhengzhou University from September 2015 to December 2016 were treated with ultrasound mediated abdominal aortic balloon occlusion. Among them, 42 cases, whose balloon occlusion time was selected before the delivery and transverse incision was taken, were group A. The other 37 cases were group B, whose timing of balloon occlusion was selected after the delivery and the uterine incision made trying to avoid the placenta or double incisions. The intraoperative blood loss, utilization of blood, and other indicators were compared between the two groups.

**Results:**

The intraoperative blood loss in groups A and B was 413.8 ± 105.9 ml and 810.3 ± 180.3 ml, and the utilization of blood products in groups A and B was 30.23% and 89.2%. The total hysterectomy rate was 2.53% (2/79), with no hysterectomies in groups A and 2 cases in group B.

**Conclusion:**

The balloon occlusion of the abdominal aorta before the delivery combined with a transverse incision is more effective.

## 1. Introduction

Pernicious placenta previa refers to a pregnant patient with a history of caesarean section with placenta previa and a 50% risk of placental implantation [1]. Pernicious placenta previa is also defined as placenta previa attached to previous cesarean delivery scars and is often associated with placenta accreta [2]. The placenta invades the superficial layer of the uterus and becomes placenta accrete. The placenta then invades the deep myometrial layer of the uterus making it placenta increta. If the placenta penetrates the wall of the uterus, reaches the uterus's serosa, and invades the adjacent organs, it is called placenta percreta [3]. Pernicious placenta previa complicated with placenta accreta/increta/percreta is a serious long-term complication of cesarean sections and often leads to unmanageable, massive hemorrhaging. This can easily lead to serious complications such as disseminated intravascular coagulation and shock [4]. For the past 10 years in China, the incidence rate of pernicious placenta previa complicated with placenta accreta/increta/percreta has gradually risen as a result of the increased use of cesarean delivery, as well as the implementation of the two-child policy [5]. Its incidence has been as high as 1/533 cases [3] and has become an important cause of postpartum hemorrhaging, intrapartum hysterectomy, and maternal mortality. In recent years, the number of patients with pernicious placenta previa complicated with placenta accreta/increta/percreta being admitted to our hospital is increasing every year. In September 2015, our team performed the first ultrasound mediated abdominal aortic balloon occlusion for the treatment of cesarean section with pernicious placenta previa [6]. A total of 79 cases were implemented in the operation until December 2016. We have found that there is a significant difference in the amount of intraoperative bleeding as the timing of abdominal aortic occlusion changes. The clinical data are summarized as follows.

## 2. Materials and Methods

### 2.1. Study Objects

#### 2.1.1. Clinical Data

A retrospective study was conducted among 119 patients with pernicious placenta previa complicated with placenta accreta who were admitted to the Obstetrics Department of the Second Affiliated Hospital of Zhengzhou University from September 1, 2015, to April 30, 2017. One case was a twin pregnancy, and the others were singleton pregnancy. These pregnant women were aged between 22 and 43 years old. 86 cases received a cesarean section for the first time, 24 cases for the second time, 7 cases for the third time, and 2 cases for the fourth time. The times of gestation termination were between 25 + 2 weeks and 39 + 2 weeks. 21 cases underwent emergency surgery because of vaginal bleeding after admission. The times of gestation termination of the remaining 98 cases were between 35 + 4 weeks and 39 + 2 weeks. Preoperative selection of abdominal aortic balloon occlusion was performed according to ultrasound examinations and past medical history scores.

#### 2.1.2. Diagnostic Criteria of Ultrasound

Specific quantification scoring was performed according to the 5 criteria for Calì et al. [7] ultrasonographic diagnosis of placental implantation: (1) loss/irregularity of the echolucent area between the uterus and placenta; (2) thinning or interruption of the hyperechoic interface between the uterine serosa and bladder wall; (3) presence of turbulent placental lacunae with high-velocity flow (>15 cm/s); (4) hypervascularity of the uterine serosa-bladder wall interface; and (5) irregular intraplacental vascularization characterized by tortuous confluent vessels across the placental width. Each item was assigned 2 points (negative 0 points, positive 2 points).

#### 2.1.3. Standard of Medical History

There was a history of cesarean section for these patients, and scoring was based on the number of times of cesarean section.

#### 2.1.4. Inclusion Criteria

The inclusion criteria were evaluated from two perspectives, medical history and ultrasound, with a total score of 16 points. 79 cases, who were evaluated as equal or more than 8 points, received the abdominal aortic balloon occlusion during the operation, so they were included as the subjects of this research group. Another 40 cases (≤8 points) were excluded.

### 2.2. Surgical Grouping

The choice of balloon dilatation time and uterine incision varies according to the experience and cognition of the surgical doctor, Among the subjects, 42 cases were regarded as group A, as the abdominal aortic occlusion time was selected before the fetal delivery, the transverse incision was selected in the lower uterine segment, and the fetus was delivered through the placenta hole. Another 37 cases were regarded as group B, with the abdominal aortic occlusion time selected after delivery of the fetus and the uterine incision was made to avoid the placenta and double incisions (first, a transverse incision was taken in the uterine body to deliver the fetus; then the abdominal aorta was blocked and the uterus suture was sutured. Then, another transverse incision was made on the lower uterine segment to deliver the placenta).

### 2.3. Surgical Methods

#### 2.3.1. Preoperative Preparation

The two groups had the same preoperative preparation, all in the general operation room. Before the operation, the abdominal aorta was scanned by the ultrasonic probe (General Company, USA) at the left axillary midline of the pregnant woman to measure its diameter, thus determining the model of Cordis balloon catheter to use (US CORDIS Company, Cordis MAXILD).

#### 2.3.2. Anesthesia

Combined spinal-epidural anesthesia was adopted during the operation.

#### 2.3.3. Balloon Catheterization of Abdominal Aorta

After anesthesia was administered, the interventional surgeon started the correct femoral artery puncture approach, and the Cordis balloon catheter was put into the abdominal aorta under the guidance of ultrasound. The catheter was placed between the lower part of the renal artery ostia and the bifurcation of the common iliac artery (avoiding the opening of the renal artery) and then fixed and attached to three-limb tubes outside the body.

#### 2.3.4. Implementation of Cesarean Section

**Figure 1:**
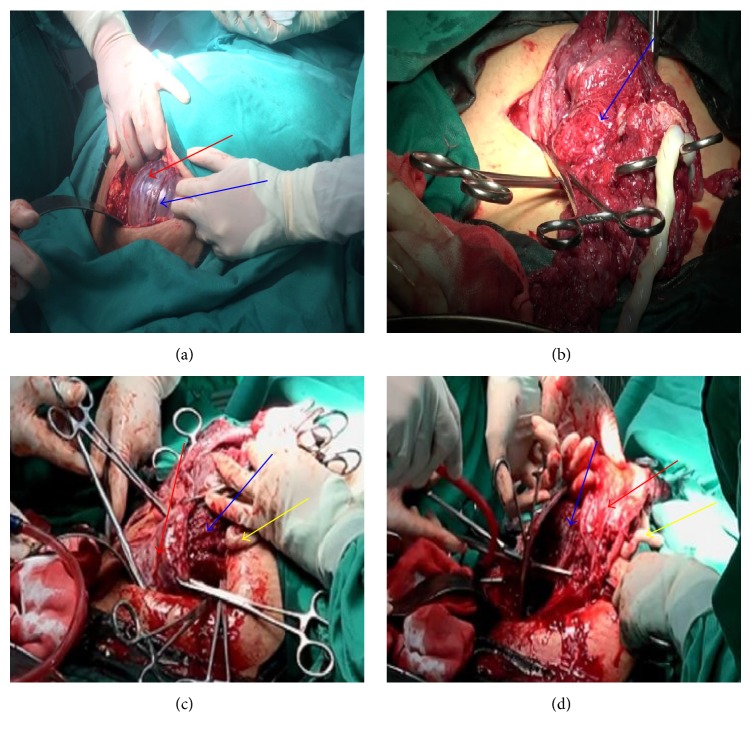
(a) Severe placenta previa with extensive penetrating accreta was found in cesarean section. Thinning or interruption of the uterine interface (red arrow), hypervascularity of the uterine interface (blue arrow). (b) The delivery of fetus through placental hole (the blue arrow is placenta). (c, d) During the operation, the placenta was cut off together with a portion of the uterus wall because of extensive penetrating accreta of the placenta (the red arrow was the lower part of the uterus where severe penetrative placenta previa was found in cesarean section, the blue arrow is the placenta, and the yellow arrow is the umbilical cord).

**Figure 2:**
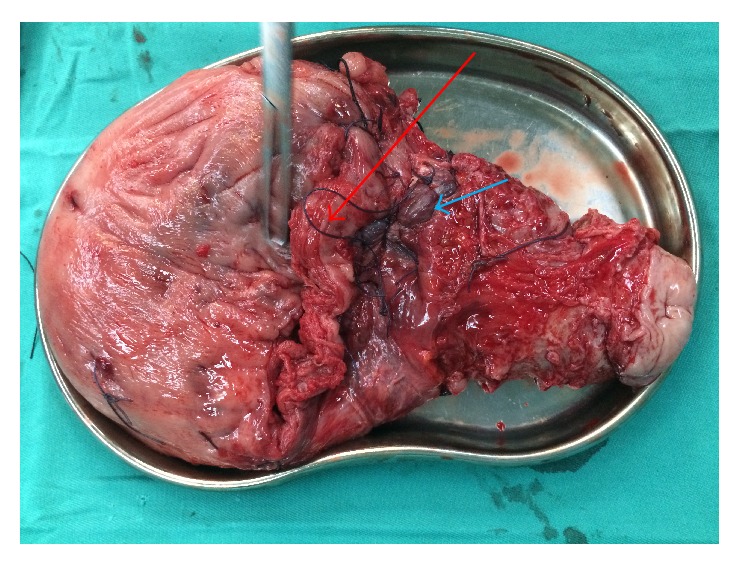
The removed uterus due to uncontrolled bleeding during the uterine double incision surgery (the red arrow for the incision of fetal delivery, and blue arrow for the placental incision).

In group A, a lower transverse incision was made in the lower segment of the uterus (the original scar site) with sufficient separation (blunt and sharp), reverse peritoneum, and pushing the bladder as far as possible (to make full preparations for the removal and repair of the uterus). Before opening the myometrium, the interventional surgeon was instructed to concurrently dilate the balloon to block the blood supply and to monitor the blood flow by ultrasonography. When the blood supply was blocked, the myometrium was cut open at the same time to enlarge the incision by hand. Then, the surgeon's right hand vertically entered the placenta at the incision site to deliver the fetus immediately (Figure 1(b)). The process from the incision of the myometrium to fetus delivery is completed in 30 seconds. In group B, the uterus incision was chosen as far as possible from the uterine body to avoid the placenta and having to make a double incision on the uterus. After opening the abdominal cavity, the uterine body should be exposed as much as possible. A transverse incision was made on the uterine body without placental attachment to open the perimetrium and muscular layer so the fetus could be delivered and the abdominal aorta could be blocked. After suturing the uterine incision, a transverse incision was made again on the lower uterine segment to deliver the placenta (Figure 2). In both groups, the umbilical cord was cut immediately after delivery and handed over the offstage. At the same time, oxytocin (Hemabate) was injected into the uterus to observe the uterine contractions and bleeding. After the contraction of the uterus, the placenta was removed with a sharp, blunt dissection. If the placenta penetrated a large area of the myometrium (Figure 1(a)), parts of the muscular layer of the penetrating part, together with the placental tissue, were cut off together (Figures 1(c) and 1(d)). Number 1 absorbable suture was used to suture the lower part of the uterus and the placental detachment surface at the mouth of cervix in an 8-character-pattern to fully stop the bleeding. Then, the balloon was closed to observe whether or not there is active bleeding. If the bleeding was not complete, the balloon could be expanded and closed again until no bleeding occurred, after which the uterus was sutured or repaired. According to intraoperative placental accreta and bleeding, a hysterectomy could be performed if necessary.

### 2.4. Observation and Follow-Up

The intraoperative bleeding, blood transfusion, hysterectomy, and the neonatal asphyxia were observed. The movement and sensation of both lower extremities were observed after operation to determine whether there were long-term complications during the 42-day review.

### 2.5. Blood Loss Statistics

According to the proportion of 1.05 g = 1 ml of blood, the weight of the gauze pad, gauze, and dressing, which were soaked by blood, was subtracted from the preoperative weight, and the volume of blood in the suction cylinder and from vaginal bleeding after operation was added. The final result was the amount of intraoperative blood loss.

### 2.6. Statistical Analysis

All data were analyzed by SPSS 21.0 software. The data description of the normal distribution was expressed by $\left(\bar{x}\pm S\right)$. The measurement data were analyzed by *t*-test and the count data were compared with the *χ*^2^ test, Fisher exact probability test, and Fisher's exact test. The results showed statistical difference in the two groups (*P* < 0.05).

## 3. Results

### 3.1. The Comparison of the General Situation between the Two Groups

There was no significant difference between A group and B group in the age, gestational age, gestational age, or previous cesarean section history (*P* > 0.05), as shown in Table 1.

### 3.2. Comparison of Intraoperative Conditions between the Two Groups

There were significant differences between the two groups in intraoperative blood loss and intraoperative blood product utilization (*P* < 0.001). There were no hysterectomies performed in group A, and there were 2 hysterectomies performed in group B. There was no significant difference between the two groups in hysterectomy rate, as shown in Table 2.

### 3.3. Complications

There was one death in group A, the cause of death being severe complications of the abdominal aortic dissection. No complications occurred in the other patients. No femoral artery thrombosis occurred in any patients (conventional low-dose heparin was used after operation), and no femoral nerve injury occurred.

### 3.4. Comparison of Maternal and Neonatal Outcomes after Operation

The condition of the newborns in both groups were evaluated by comparing the Apgar Scores in 1 min and 5 min after the delivery of the fetus, and the results were not statistically significant between the two groups (*P* > 0.05), as shown in Table 3 (the Apgar scores at 10 min were mostly 10 points, so comparisons were no longer needed).

### 3.5. Follow-Up Results

Among the 79 cases, 72 cases were followed up, and 6 cases were lost. The follow-up period ranged from 6 weeks to one year. Of the 72 patients, 2 cases in group B received interventional therapy because of postoperative rebleeding. One case in group A died of massive retroperitoneal hematoma formed by the abdominal aortic dissection the following day. All patients were discharged without any abnormalities in their movements or sensations at the time of discharge. At 6 weeks after the operation, all patients had *β*-HCG < 5 mIU/ml. The lochia in 37 cases was not completely clean, of which ultrasounds of 6 cases indicated that there were strong and heterogeneous echo in the palace cavity. The lochia in 35 cases was completely clean, and ultrasounds indicated that a small amount of fluid echo can be seen in the uterine cavity. 16 cases displayed recurrent menstruation, and the menstrual volume and menstrual period had no obvious changes when compared with preoperation levels.

## 4. Discussion

Pernicious placenta previa complicated with placenta increta/percreta can lead to serious long-term complications after a cesarean section. After a cesarean section, endometrial damage, poor scar healing at the incision, endometrial defects, and other factors can lead to villi and the placenta invading the muscularis and serosa layer, and possibly the bladder and pelvic wall, which are susceptible to forming placenta previa and placenta accreta [8]. Placenta accreta and intractable hemorrhaging are two characteristics of pernicious placenta previa. Angstmann et al. [9] pointed out that the average amount of bleeding during the cesarean section of patients with pernicious placenta previa can be as high as 3000–5000 mL, and the hysterectomy rate has been reported to be as high as 55%–75% [10, 11]. Removing the uterus to save the mother's life is difficult for most Chinese patients and their families to accept due to the loss of fertility. In order to guarantee safety for the mother and newborns, the surgical treatment of pernicious placenta previa complicated with placenta increta/percreta requires a treatment team composed of a senior physician with experience in placenta implantation treatment, anesthesiologist, pediatricians with experience in preterm infants, operation room personnel, blood bank personnel, and so on. If vascular occlusion is needed, an intervention team is also required. Ultrasounds are used in our hospital. Therefore, the senior doctors with diagnostic experience in the ultrasound department are also important team members. Our obstetrics department has two chief physicians with the ability to diagnose and treat the disease, so patients are given to one of the two physicians on a randomized basis in combination with the patient's wishes. The two doctors have different surgical experiences and different surgical methods. One doctor chooses to block the abdominal aorta before the fetus is delivered, make a lower uterine segment transverse incision, and use the placenta hole to deliver the fetus. Another doctor chooses to block the abdominal aorta after the fetus is delivered, and the uterine incision is made trying to avoid the placenta and making a double incision. As a result, the patients are automatically divided into two groups according to the doctor's different surgical methods.

In order to standardize and guide the diagnosis and treatment of clinical placenta accreta, the Chinese Medical Association Perinatal Medicine Branch organized experts to write the Clinical Guidelines for Placenta Accreta in 2015 [3]. According to the guidelines, ultrasound is the most commonly used method for predicting placental implantation. When an ultrasound indicates that there is structural disorder of the placental site, diffuse or focal lacunar flow occurs in the placenta, the normal hypoechoic areas of the placenta are thinned or gone, or the perimetrium-bladder junction is rich in blood flow, the sensitivity of placental implantation is 83% (95% CI: 77%–88%), and the specificity is 95% (95% CI: 93%–96%). When the local hospital finds more than one in an ultrasound image, the obstetrician will transfer the patient from the local hospital to a higher level hospital for diagnosis and treatment. However, the study by Sumigama et al. [12] showed that only 37% of cases of pernicious placenta previa were associated with placenta increta/percreta. In our hospital, the rate of pernicious placenta previa complicated with placenta accreta is as high as 66.39% (79/119). This is because 94.96% of our patients (113/119) were transferred from other cities and counties in the Henan Province. If a patient was transferred, it was because she was highly suspected to have pernicious placenta previa complicated with placenta accreta, so our high placental implantation rate was associated with the initial screening at the time of referral.

There are many methods for treating dangerous placenta previa. At present, many studies focus mainly on internal iliac artery embolization (IIAE), uterine artery embolization (UAE), abdominal aortic balloon occlusion, and so on [12–17]. There is an especially heavy focus on abdominal aortic balloon occlusion because although there are no uniform guidelines, there is a great advantage in controlling intraoperative bleeding and preserving the uterus. Because of the limitations of X-ray mediated occlusion, Zhang et al.'s [15] study showed a hysterectomy rate as high as 18%, mainly because of the large amount of postoperative bleeding. Therefore, Zhang thought that it was related to an incomplete occlusion or occlusion failure. We believe that ultrasound mediated occlusion can solve this problem. Our study has suggested that the diameter of the abdominal aorta can be measured before the ultrasound, and then a balloon catheter that is consistent with the diameter can be chosen to achieve a better occlusion effect. Intraoperative blood pressure can also be checked by ultrasonography.

Many experts believe that the abdominal aortic obstruction should be delivered after the fetus [13–17]. The so-called “abdominal aorta balloon occlusion” is to create a “no blood” environment for the operation, which can reduce bleeding during the operation and also provide convenient conditions for suture hemostasis. In Figure 1(a), the placenta is inserted into the lower part of the anterior wall of the uterus and even penetrates the myometrium to the surface of the uterus. In this case, if the timing of the balloon occlusion is selected for after fetal delivery, then massive bleeding will occur when the uterus is cut to deliver the fetus. Thus, the best opportunity to control hemorrhaging of the uterus is missed. If the timing of the balloon occlusion is selected for before the fetal delivery, the uterine bleeding from the time of the uterine incision to the fetal delivery can be effectively controlled (Figure 1(b)). It can also effectively control the uterine bleeding before and after placental abruption and provide convenient conditions for suture hemostasis. Our data showed that the amount of bleeding in group A, which was blocked before the delivery of fetus, was (413.8 ± 105.9) ml, which was significantly lower than group B (810.3 ± 180.3) ml, which was blocked after delivery of fetus. Also, the intraoperative use of blood products in group A was also significantly less than in group B, and the differences were statistically significant (*P* < 0.001).

Due to the balloon dilation before delivery of the fetus blocking the blood supply to the placenta, the speed of the fetal delivery determines the birth status of the fetus. According to our experience, because the abdominal aorta balloon occlusion blocks the blood supply to the placenta, if the fetus can be delivered and the cord cut in a few seconds after emerging through the placenta hole, the fetus will quickly establish its own blood circulation after delivery. Thus, the fetus in the uterus undergoes only a few seconds of ischemia, which will not affect its Apgar score. In group A, the differences in Apgar score 1 min and 5 min after delivery when compared with group B were not statistically significant (*P* = 0.126,  *P* = 0.478). Although patients in group B were selected to undergo abdominal aortic occlusion after delivery, the incision avoided the placenta as much as possible, thus avoiding hemorrhaging of the placenta and uterus before and after delivery and the neonatal Apgar score was not affected.

The ACOG committee [18] of the United States believes that the location of the incision of the uterus depends on the location of the placenta. In principle, the placenta, or at least the main part of the placenta, should be avoided in the incision. Our experience has shown that if the incision is made high, the placental hole is avoided and uterine incision bleeding is relatively less, but the distance between the incision and the mouth of cervix is far. This leads to difficulty controlling the bleeding of the placental peeling surface and in the mouth of the cervix. If the bleeding is not controlled, the hysterectomy rate will also increase. Therefore, we believe that if the abdominal aorta is blocked before the delivery, a transverse incision of the lower uterine segment is the best choice.

Our data have shown that 2 patients underwent hysterectomies, with a hysterectomy rate of 2.53% (2/79), which is below Zhang et al.'s [15] report of 18%. A report by Liu and Zhao [14] suggested that there were no hysterectomies in 230 cases of placenta previa complicated with extensive placenta accrete. In this study, ultrasounds guided the application of the appropriate balloon catheter diameter, which can reduce the incidence rate of incomplete occlusions. This can effectively reduce uterine bleeding before and after placental abruption and provide convenient conditions for surgical suture hemostasis. In addition, according to the size and depth of the placental implantation, different treatment methods were used. Therefore, our effective interventional approach and surgical and suture techniques for preserving the uterus have significantly reduced our hysterectomy rate.

Abdominal aortic occlusion is the temporary occlusion of the blood supply of the pelvic cavity and lower extremities during a cesarean section. Bodner et al. [19] believe that the occlusion timing is the safest within 30 minutes of delivery so as to not cause ischemic necrosis of the pelvic organs and lower extremities. The study also showed that the 30-minute period is sufficient to control uterine bleeding. There are a few reports of interventional complications associated with balloon occlusion of the abdominal aorta. According to Liu and Zhao's study [14], 2 of 230 cases with abdominal aortic balloon occlusion developed femoral artery thrombosis in their lower limbs. None of our cases developed femoral artery thrombosis or femoral nerve injuries, which can be credited to our routine use of small doses of heparin. Unfortunately, one case of the intervention group developed severe interventional complications relating to the abdominal aortic dissection. This reminds us of the necessity of performing an ultrasound rescan of the abdominal aorta and femoral artery at the end of the operation.

During the caesarean section in patients with pernicious placenta previa complicated with placenta increta/percreta, our study demonstrated that a balloon occlusion of the abdominal aorta before the delivery of the fetus combined with a transverse incision on the lower uterine segment, can effectively control the amount of intraoperative blood loss and reduce the utilization rate of intraoperative blood products. This practice can also significantly reduce the hysterectomy rate and have no effect on the neonatal Apgar score and vital signs.

## Figures and Tables

**Table 1:** The comparison of the general situation between the two groups.

Group	Cases	Age (years old)	Gravidity	Previous cesarean section times	Gestational weeks (week)	Cesarean section hemorrhage
Group A	42	31.8 ± 5.2	3.5 ± 1.5	1.1 ± 0.3	35.5 ± 2.8	1.1 ± 0.3
Group B	37	31.6 ± 3.6	3.4 ± 1.5	1.4 ± 0.6	35.5 ± 3.0	1.4 ± 0.6
*t*/*χ*^2^ value		0.208	0.107	−1.717	0.055	−1.717
*P* value		0.836	0.915	0.092	0.957	0.092

**Table 2:** Comparison of intraoperative conditions between the two groups.

Group	Cases	Intraoperative blood loss (ml)	The use of blood products		Hysterectomy	
			Yes (%)	No (%)	Yes (%)	No (%)
Group A	42	413.8 ± 105.9	13 (31.0)	29 (69.0)	0 (0.0)	42 (100.0)
Group B	37	810.3 ± 180.3	32 (88.9)	4 (11.1)	2 (5.6)	34 (94.4)
*t*/*χ*^2^ value		−11.593	26.658		—	
*P* value		<0.001	<0.001		0.210^*∗*^	

*Note.* ^*∗*^Fisher's exact test.

**Table 3:** Comparison of maternal and neonatal outcomes after operation.

Group	Cases	Apgar score in 1 min	Apgar score in 5 min
Group A	42	8.5 ± 3.1	8.9 ± 2.6
Group B	37	9.4 ± 1.9	9.3 ± 2.3
*t*/*χ*^2^ value		−1.549	−0.714
*P* value		0.126	0.478

## References

[1] Xie X., Gou W. L. (2013). Obstetrics and Gynecology. *People’s Medical Publishing House*.

[2] Chen Z., Li J., Shen J., Jin J., Zhang W., Zhong W. (2016). Direct puncture embolization of the internal iliac artery during cesarean delivery for pernicious placenta previa coexisting with placenta accreta. *International Journal of Gynecology and Obstetrics*.

[3] (2015). Guidelines for the diagnosis and treatment of placenta accreta. *Chinese Journal of perinatal medicine*.

[4] Wang J. M., Shi F. X., Wang F. (2015). Application of immediate artery embolization after fetal disengagement in rescue of postpartum hemorrhage induced by implantable dangerous placenta previa. *Maternal Child Health Care of China*.

[5] Liu Y.-H., Teng J.-Y., Zheng Z.-X. (2014). Emodin regulates glucose uptake by activating sirtl in 3T3-L1 adipocytes. *Journal of Nanjing University of Chinese Medicine*.

[6] Yang K. L., Zhang Z. R., Zhu B. J., Zhu H. J. (2016). Ultrasound mediated lower abdominal aorta balloon block on research and application of timing of surgery in pernicious placenta previa during childbirth. *life Science Journal*.

[7] Calì G., Giambanco L., Puccio G., Forlani F. (2013). Morbidly adherent placenta: evaluation of ultrasound diagnostic criteria and differentiation of placenta accreta from percreta. *Ultrasound in Obstetrics and Gynecology*.

[8] Garmi G., Salim R. Epidemiology, etiology, diagnosis, and management of placenta accreta.

[9] Angstmann T., Gard G., Harrington T., Ward E., Thomson A., Giles W. (2010). Surgical management of placenta accreta: a cohort series and suggested approach. *American Journal of Obstetrics and Gynecology*.

[10] Hull A. D., Moore T. R. (2011). Multiple Repeat Cesareans and the Threat of Placenta Accreta: Incidence, Diagnosis, Management. *Clinics in Perinatology*.

[11] Zhou Z. C., Wang C. H., H. X (2011). Application of early ligation of bilateral uterine artery superior branch in implantable dangerous placenta previa. *Journal of Practical Obstetrics and Gynecology*.

[12] Sumigama S., Itakura A., Ota T. (2007). Placenta previa increta/percreta in Japan: A retrospective study of ultrasound findings, management and clinical course. *Journal of Obstetrics and Gynaecology Research*.

[13] Wang Y. L., Duan X. H., W X. (2015). Temporary aortic balloon occlusion in management of cesarean section for pernicious placenta previa/accreta. *Journal of Practical Radiology*.

[14] Liu C., Zhao X. L. (2016). The Application of Temporary Ballon Occlusion of the Abdominal Aorta in Patients with Pernicious Placenta Previa and Placenta Accreta. *Journal of Practical Obstetrics and Gynecology*.

[15] Zheng Q. Q., Liu T., Xie K. Q. (2016). Anti-influenza virus activities of garlic oil in mice model *in vivo*. *Journal of Shandong University (Health Science)*.

[16] Zhou Y., Yang Y., Huang Y. (2014). The Application of temporary ballon occlusion of the abdominal aorta in pernicious placenta previa. *Fujian Medical Journal*.

[17] Gan B. (2015). Clinical analysis of distal abdominal preoperative balloon occlusion for the treatment of dangerous placenta previa. *Chinese and Foreign Medical Research*.

[18] American College of Obstetricians and Gynecologists (2013). ACOG committee opinion no. 559: cesarean delivery on maternal request. *Obstetrics and gynecology*.

[19] Bodner L. J., Nosher J. L., Gribbin C., Siegel R. L., Beale S., Scorza W. (2006). Balloon-assisted occlusion of the internal iliac arteries in patients with placenta accreta/percreta. *CardioVascular and Interventional Radiology*.

